# Supplementary figures and images for: Research on the characteristics and influencing factors of the Beijing-Tianjin-Hebei urban network structure from the perspective of listed manufacturing enterprises
Source: PLoS One. 2023 Jan 20;18(1):e0279588. doi: 10.1371/journal.pone.0279588 (PMC9858376; doi:10.1371/journal.pone.0279588)

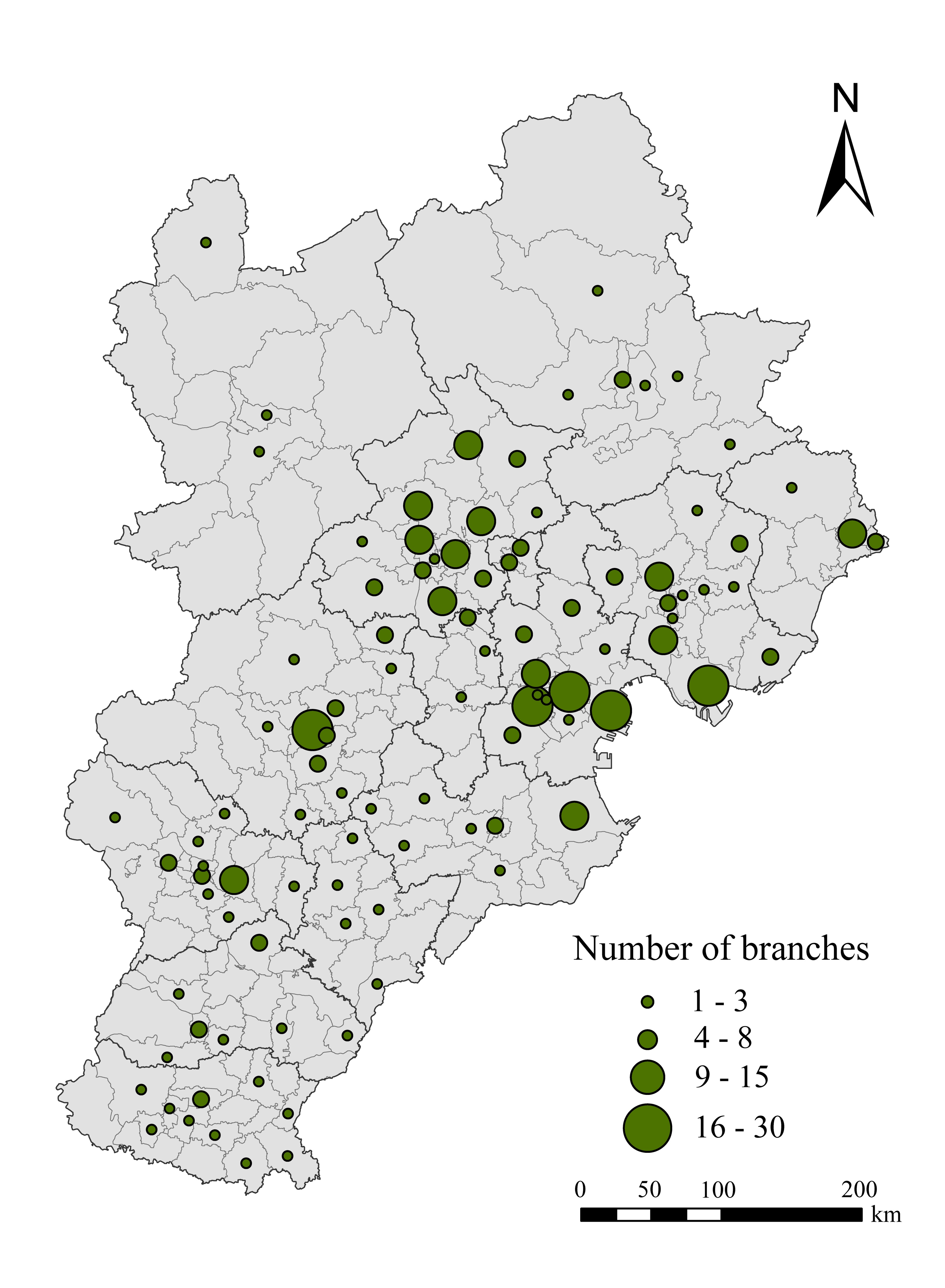

Supplement: S1 Fig — The figure shows the spatial distribution of the listed manufacturing enterprise headquarters and branches in the Beijing-Tianjin-Hebei region. (ZIP) [file pone.0279588.s001.zip › S1 Fig. Data of the listed manufacturing enterprises/S1 Fig. Number of branches..tif]

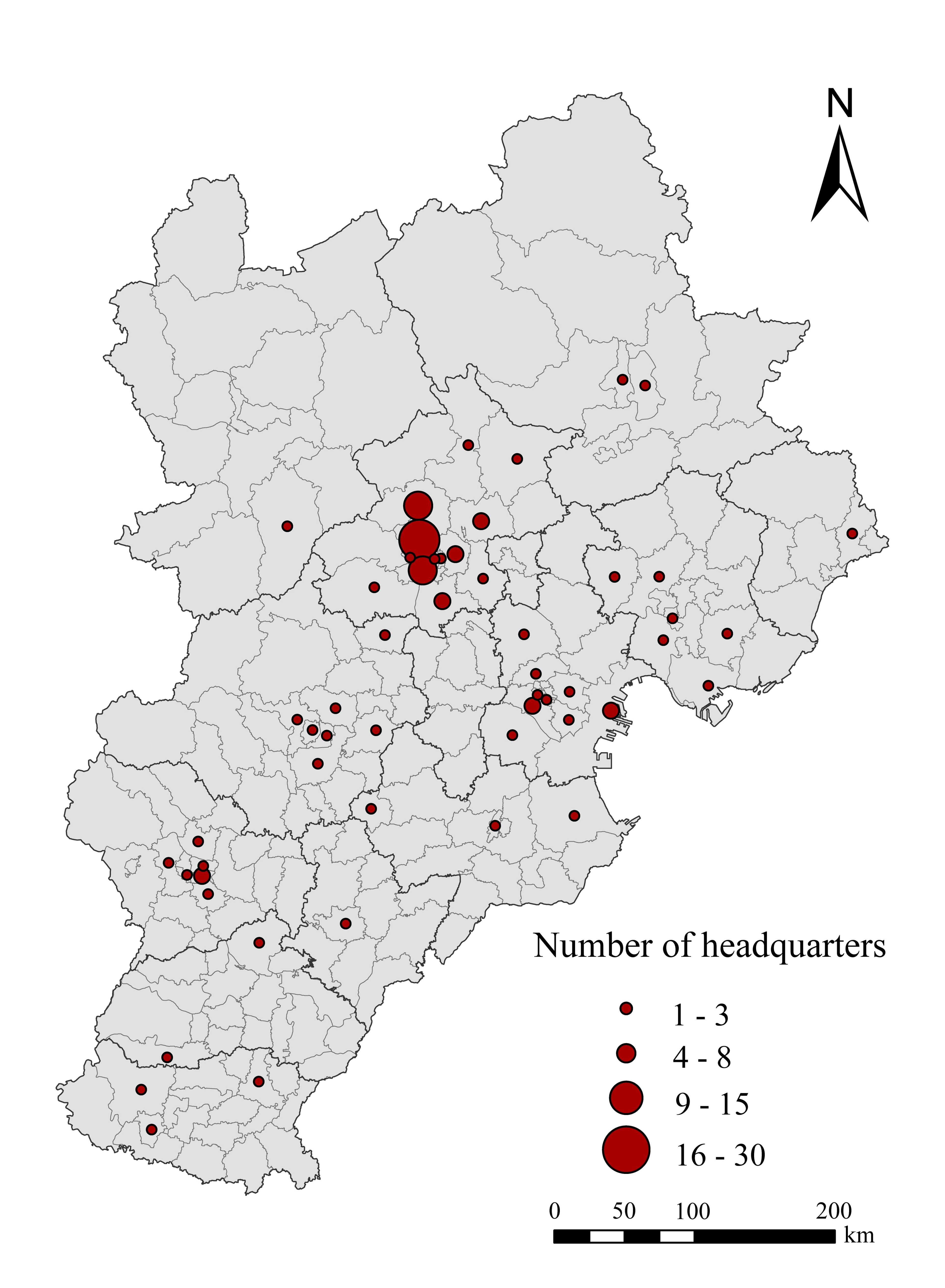

Supplement: S1 Fig — The figure shows the spatial distribution of the listed manufacturing enterprise headquarters and branches in the Beijing-Tianjin-Hebei region. (ZIP) [file pone.0279588.s001.zip › S1 Fig. Data of the listed manufacturing enterprises/S1 Fig. Number of headquarters..tif]

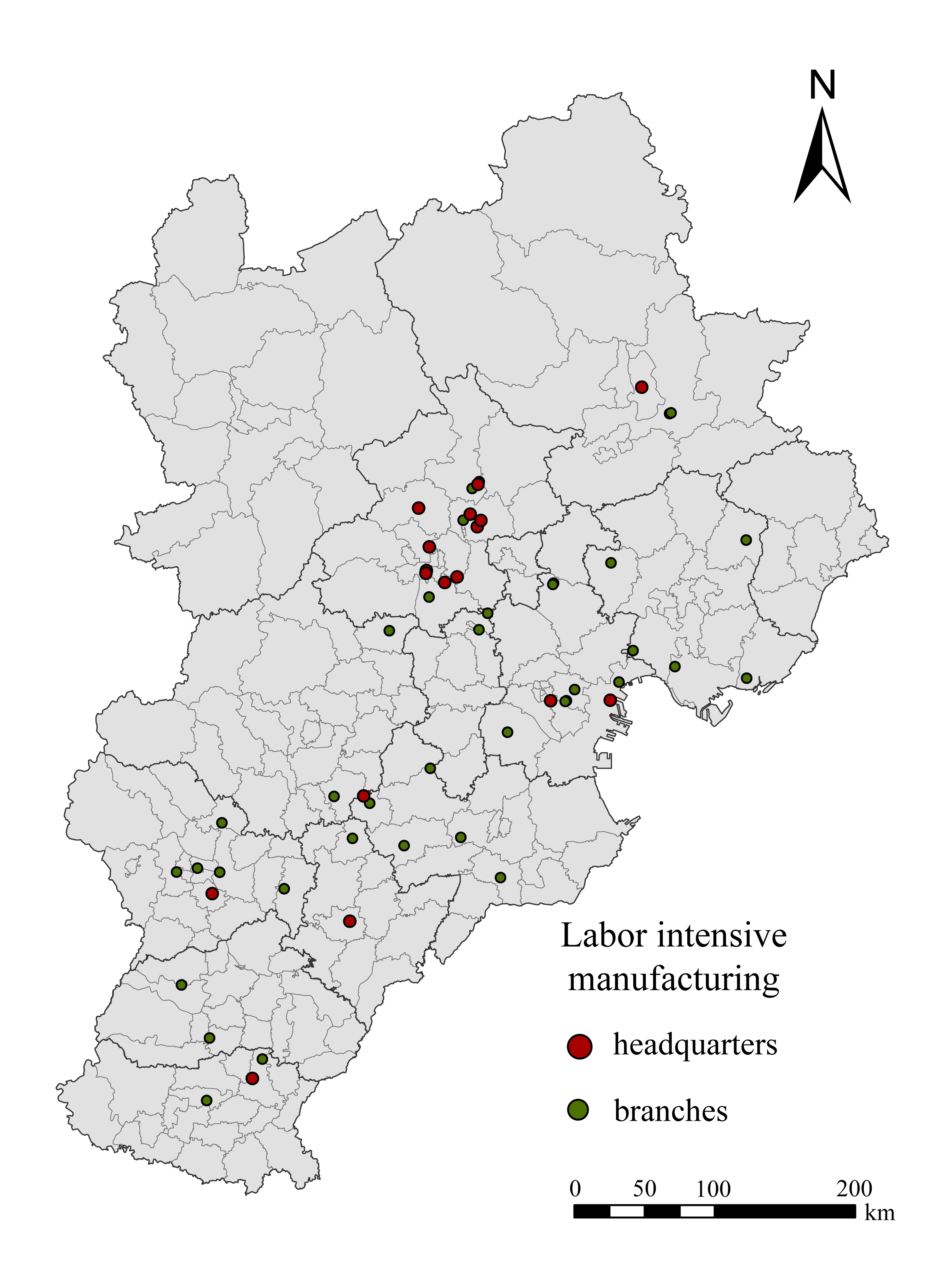

Supplement: S2 Fig — The figure shows the spatial distribution of technology-intensive, resource-intensive, labor-intensive and capital-intensive manufacturing enterprises in the Beijing-Tianjin-Hebei region. (ZIP) [file pone.0279588.s002.zip › S2 Fig. Data of the listed manufacturing enterprises by type/S2 Fig. Labor-intensive manufacturing.tif]

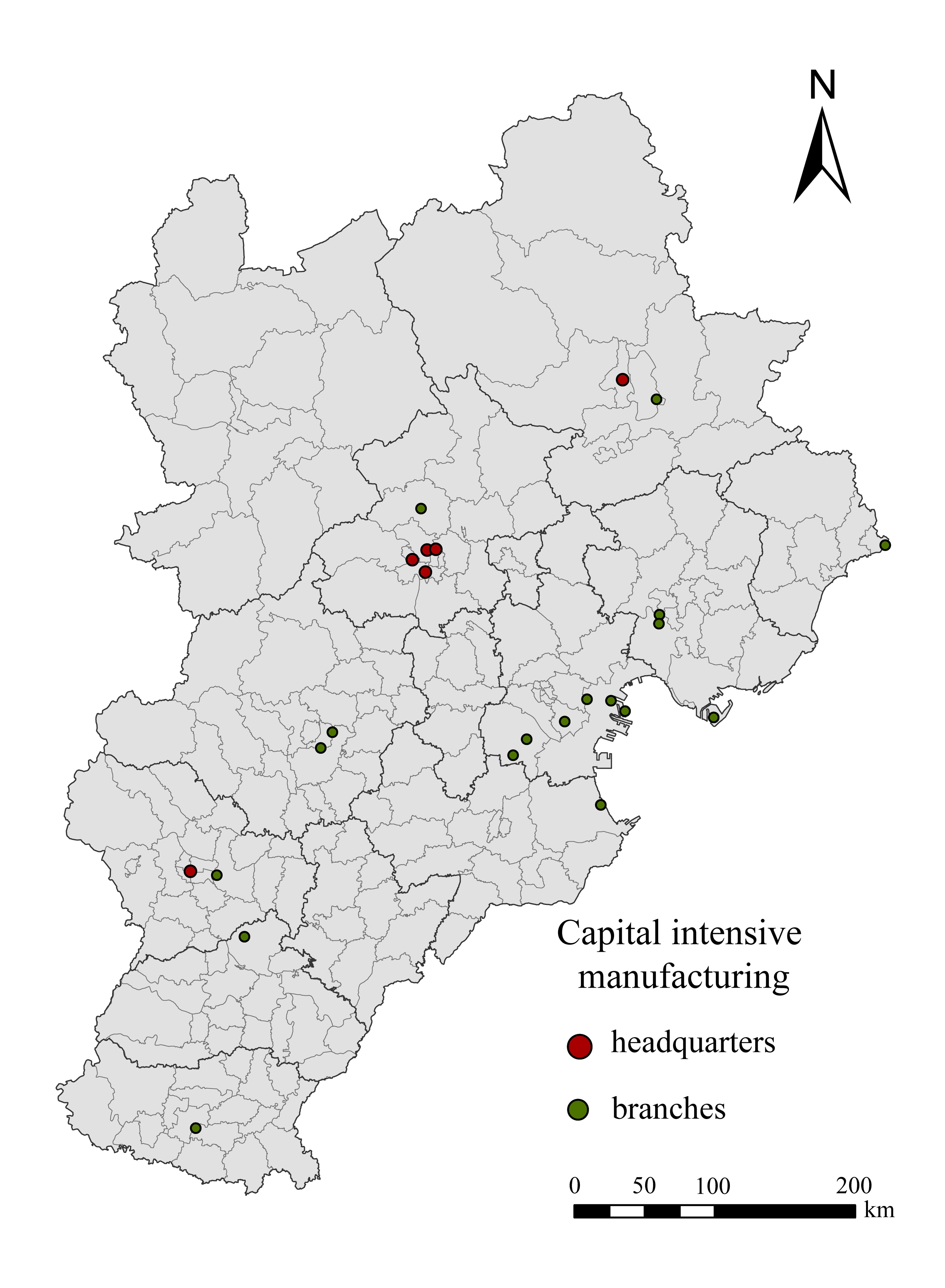

Supplement: S2 Fig — The figure shows the spatial distribution of technology-intensive, resource-intensive, labor-intensive and capital-intensive manufacturing enterprises in the Beijing-Tianjin-Hebei region. (ZIP) [file pone.0279588.s002.zip › S2 Fig. Data of the listed manufacturing enterprises by type/S2 Fig. Capital-intensive manufacturing.tif]

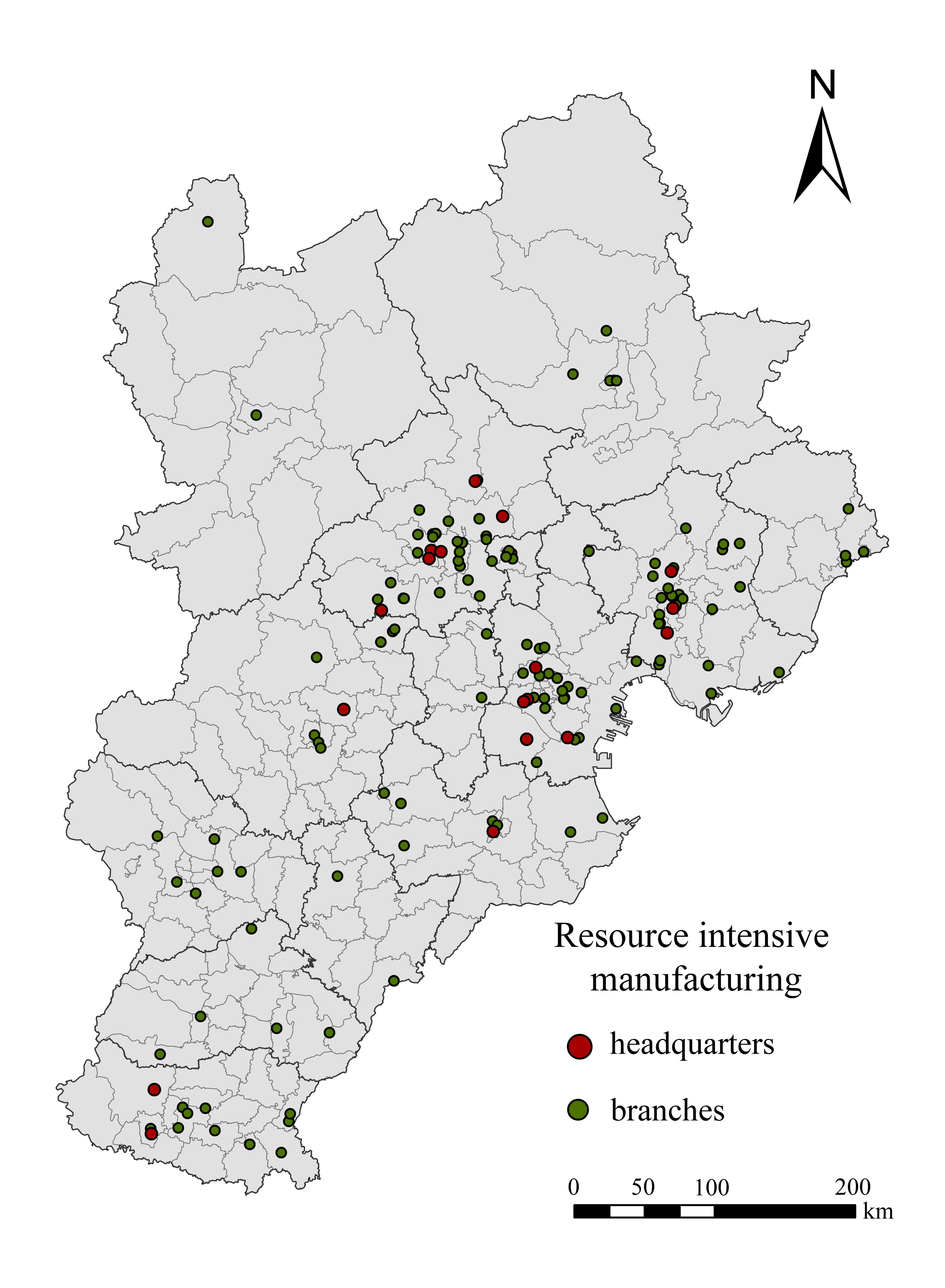

Supplement: S2 Fig — The figure shows the spatial distribution of technology-intensive, resource-intensive, labor-intensive and capital-intensive manufacturing enterprises in the Beijing-Tianjin-Hebei region. (ZIP) [file pone.0279588.s002.zip › S2 Fig. Data of the listed manufacturing enterprises by type/S2 Fig. Resource-intensive manufacturing.tif]

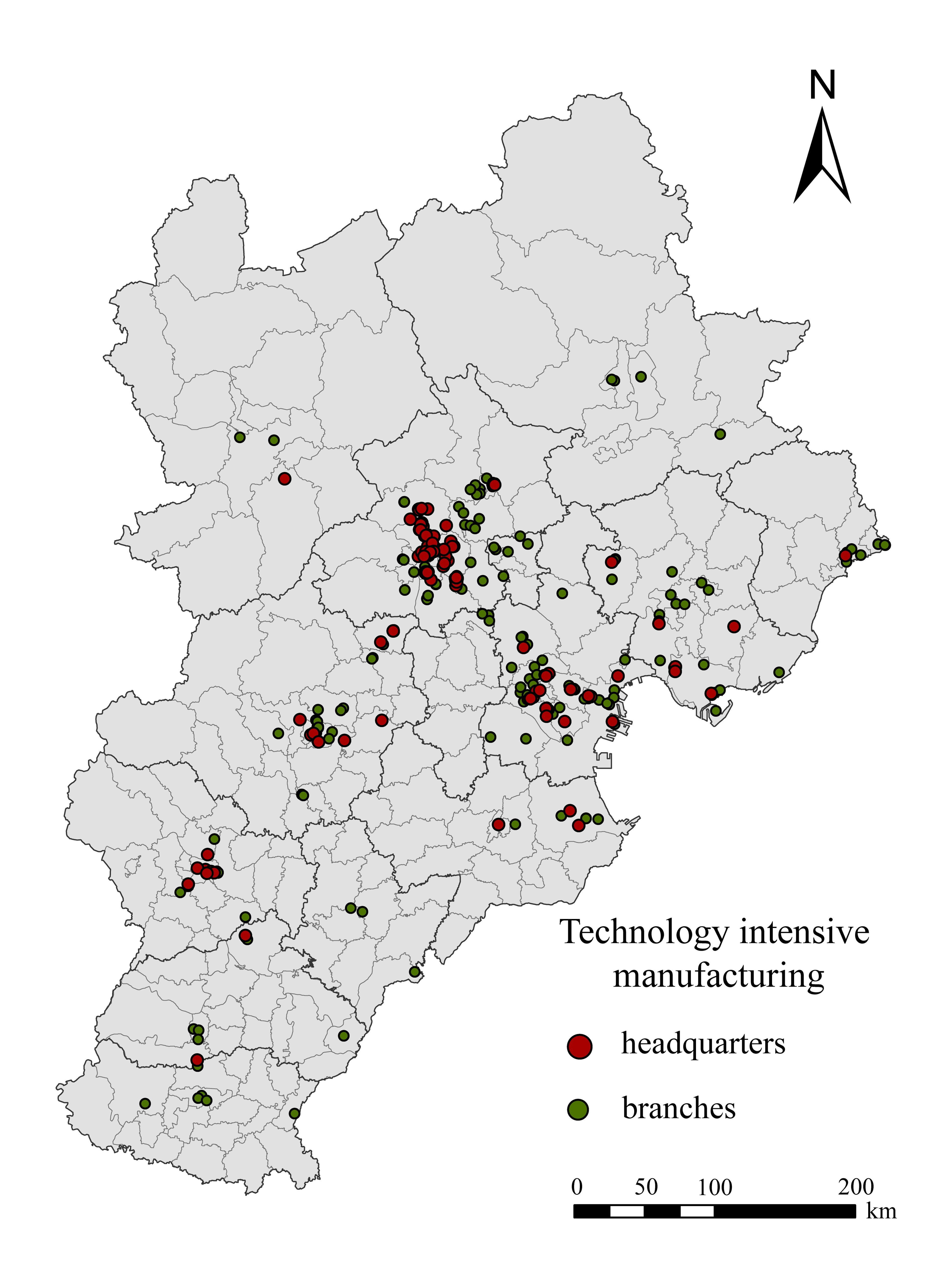

Supplement: S2 Fig — The figure shows the spatial distribution of technology-intensive, resource-intensive, labor-intensive and capital-intensive manufacturing enterprises in the Beijing-Tianjin-Hebei region. (ZIP) [file pone.0279588.s002.zip › S2 Fig. Data of the listed manufacturing enterprises by type/S2 Fig. Technology-intensive manufacturing.tif]

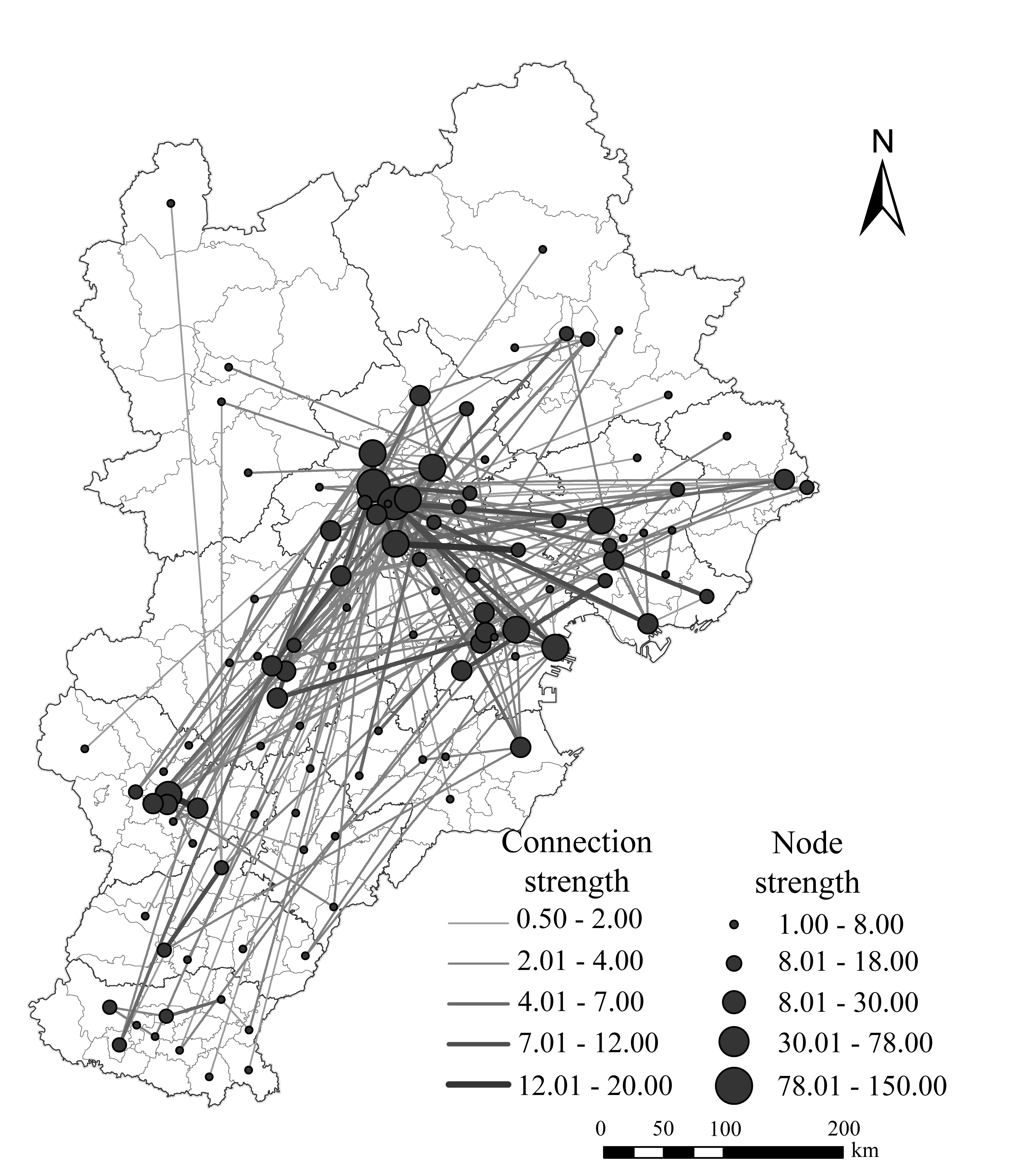

Supplement: S3 Fig — The figure shows the spatial pattern of urban networks at the prefecture and district levels in the Beijing-Tianjin-Hebei region. (ZIP) [file pone.0279588.s003.zip › S3 Fig. Connection strength and node strength data/S3 Fig. Spatial pattern of urban network at county level..tif]

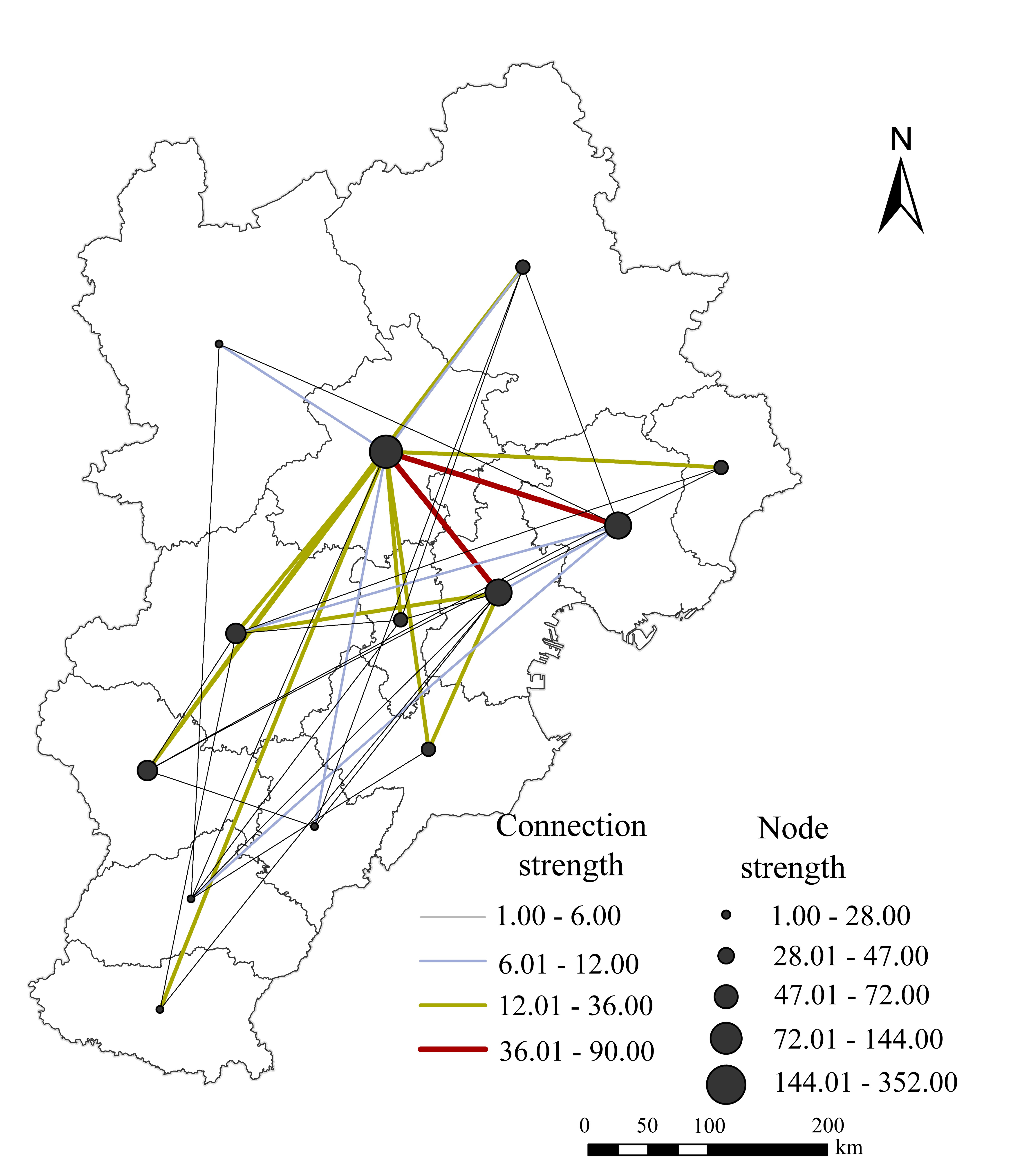

Supplement: S3 Fig — The figure shows the spatial pattern of urban networks at the prefecture and district levels in the Beijing-Tianjin-Hebei region. (ZIP) [file pone.0279588.s003.zip › S3 Fig. Connection strength and node strength data/S3 Fig. Urban network spatial pattern at prefecture level..tif]

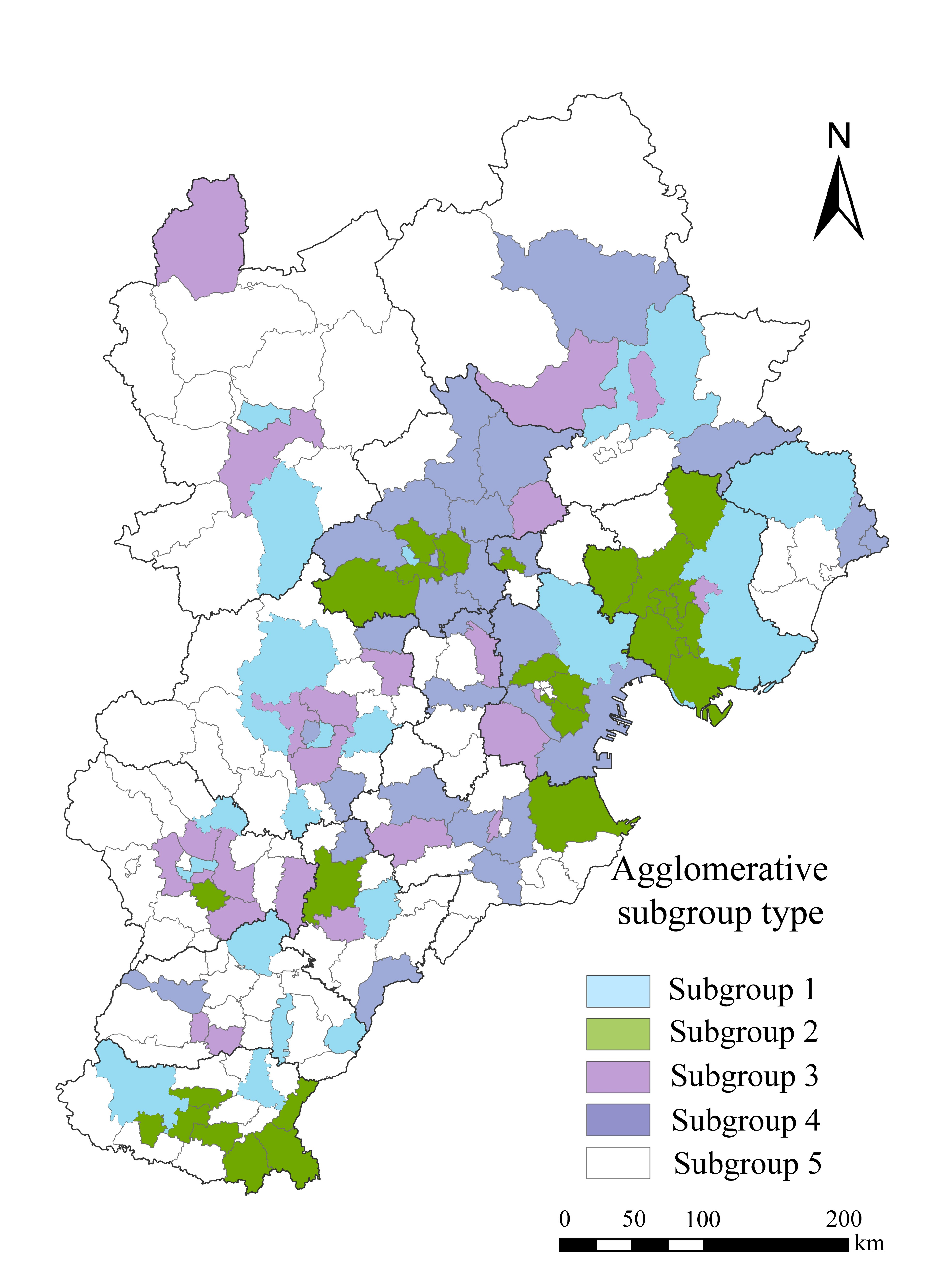

Supplement: S4 Fig — The figure shows the spatial distribution of subgroups at the district and county levels in the Beijing-Tianjin-Hebei region. (ZIP) [file pone.0279588.s004.zip › S4 Fig. Aggregate subgroup hierarchical data/S4 Fig. Agglomeration subgroup map of urban network ..tif]
