# Supplementary material for: Research on the characteristics and influencing factors of the Beijing-Tianjin-Hebei urban network structure from the perspective of listed manufacturing enterprises
Source: PLoS One. 2023 Jan 20;18(1):e0279588. doi: 10.1371/journal.pone.0279588 (PMC9858376; doi:10.1371/journal.pone.0279588)
Supplement: Attachment — Submitted filename: Response to Reviewers.docx [file pone.0279588.s006.docx]

Dear Academic Editors and Reviewers:

Thank you for your letter and for the reviewers’ comments concerning our manuscript entitled “Research on the characteristics and influencing factors of Beijing Tianjin Hebei urban network structure from the perspective of listed manufacturing enterprises” .

First of all, we are encouraged that the reviewers found the topic of our article interesting and practical, and relevant for promoting regional integration in China. Secondly, we would like to thank the reviewers for their time and effort in reviewing the manuscript and for their constructive comments on our manuscript, as well as the editorial team for their hard work. We have carefully considered the reviewers' comments and made some changes to the manuscript to the best of our ability. Below is a response to the points raised by each reviewer and an updated manuscript is provided to address each reviewer's concerns.

**Reviewer #1:**

1. *It is better to supplement the analysis of spatial correlation characteristics between parent companies and subsidiaries of different types of manufacturing enterprises.*
   **Response:**

We thank the reviewers for raising this issue, and we have added the spatial structure of urban networks of subtypes of listed manufacturing enterprises to lines 323-351 of the manuscript, and added graphical information to facilitate a more intuitive response to the spatial association characteristics among the sub-parent companies of different types of manufacturing enterprises.

**Fig. 7. Spatial distribution of the urban network of the listed manufacturing enterprises by type. (A) Technology-intensive manufacturing; (B) Resource-intensive manufacturing; (C) Labor-intensive manufacturing; and (D) Capital-intensive manufacturing.**

2. *It is suggested to arrange the contents according to the distribution characteristics of enterprises, spatial correlation analysis, network characteristics analysis, influencing factors, etc.*

**Response:**

- The distribution characteristics of enterprises are described in lines 182-230 of the manuscript.
- We believe that spatial association analysis and network characteristics are inseparable, so we describe these two parts in the chapter of "Spatial Structure Analysis of Beijing-Tianjin-Hebei City Network". The node characteristics and overall network characteristics of the city network systematically analyze the spatial association of Beijing-Tianjin-Hebei districts and counties (lines 238-295 of the manuscript), and then describe the characteristics of the city network in detail by level, type and molecular group (lines 296-379 of the manuscript).
- The final influencing factors are analyzed in lines 380-479 of the manuscript.

**Reviewer #2:**

1. *Although the introduction section of the paper mentioned the construction of urban network by talking affiliation model, the reasons and significance of choosing listed manufacturing industry are not clearly discussed. More elaboration should be given to echo the title of this paper.*

**Response:**

We appreciate the reviewers' careful reading of the text. Based on his/her suggestions, we have added the reasons and significance of the choice of listed manufacturing companies in the introduction section of the manuscript, in lines 88-91 of the full text.

2. *The dividing evidences of hierarchical network are not clear enough. More elaboration are reqiured.*

**Response:**
The issue you raised is very worthy of our consideration. The previous ranking of connection strength carries its own subjective idea and the basis of division is unclear, after referring to relevant literature and considering the number of connections in the city network of listed manufacturing enterprises in Beijing, Tianjin and Hebei, the hierarchical network is re-divided. The strength of connections between districts and counties in the network is ranked from high to low, and the top 10%, top 10%~50% and top 50%~100% network connection pairs are extracted respectively. The purpose of this division is to simplify the network analysis and facilitate a clearer description of the network characteristics at each level. For details, see lines 296-322 of the manuscript.

**Fig. 6. Hierarchical pattern of the urban network at the county level. (A) Top 10% of network contacts; (B) Top 10~50 % of network contacts; and (C) Top 50~100 % of network contacts.**

3. *Although rich literature has been reviewed in this study, there is few dialogue between the empirical findings and literature. More theoretical discussion are suggested to be given in this paper, so as to highlight the theoretical contribution of this paper.*

**Response:**

Based on reviewers' suggestions, we have added a dialogue with the literature in the manuscript.

- Page 17, lines 290-293, cites Sun Zhijing's original work, which compares and analyzes manufacturing firms in the Yangtze River Delta region with those in the Beijing-Tianjin-Hebei region.

References are: 39. Sun Zhijing. Research on urban network structure in the Yangtze River Delta region based on multiple perspectives[D]. East China Normal University, 2020. <https://doi.org/10.27149/d.cnki.ghdsu.2020.000422>

- Page 18, lines 315-319, cites Yu Qian's findings based on inter-firm fund transaction data to study the intra-Beijing-Tianjin-Hebei network structure, which validates this paper's Beijing-Tianjin-Hebei network structure.

References are: 40.Yu Qian.Research on the evolution of the internal network structure ofthe three major urban agglomerations in China[D].Fujian Normal University, 2021. <https://doi.org/10.27019/d.cnki.gfjsu.2021.000573>

4. *Professional language editing is required to express the ideas accurately.*

**Response:**

This section was revised by ourselves, and then we consulted experts and scholars in related fields for guidance on the content of the article; we do not list the changes here, but in the revised document has been marked out.

The above is our response to your valuable questions and suggestions, thank you very much for your patience! Your patience and quality suggestions have helped us a lot and we have learned a lot in improving our paper. Thank you again! We wish you good health and happiness in life!

Author: Wang Wengang, etc.
